# Carbon dots with light-responsive oxidase-like activity for colorimetric detection of dopamine and the catalytic mechanism

**DOI:** 10.3389/fchem.2023.1288418

**Published:** 2023-10-12

**Authors:** Zhenzhen Jia, Yuna Liu, Liangliang Cheng, Zhichao Deng, Mingzhen Zhang, Hang Tuo

**Affiliations:** ^1^ Department of Hepatobiliary Surgery, The First Affiliated Hospital of Xi’an Jiaotong University, Xi’an, Shaanxi, China; ^2^ School of Basic Medical Sciences, Xi’an Jiaotong University Health Science Center, Xi’an, Shaanxi, China

**Keywords:** carbon dots, light-responsive oxidase-like activity, colorimetry, dopamine, surface groups

## Abstract

**Introduction:** Dopamine is one of the most significant neurotransmitters and plays an important role in the management of cognitive functions such as learning, memory, and behavior. The disorder of dopamine is associated with many major mental diseases. It is necessary to develop selective methods for the detection of dopamine.

**Methods:** In this work, carbon dots (CDs) were synthesized by a solvothermal route using glutathione, L-histidine, and formamide as sources.

**Results:** Under light irradiation, The CDs convert dissolved oxygen to singlet oxygen (^1^O_2_), which could oxidize TMB. When reduced dopamine was present, it suppressed the catalysis of CDs, then the absorption of the CDs-coupled TMB complex at 652 nm was diminished. Furthermore, it was revealed that the surface groups including hydroxyl, amino, carbonyl, and carboxyl groups of CDs were related to their light-responsive catalytic activity by surface modification. In the range of 0.5-15 μM, the CDs could afford a LOD of 0.25 μM for dopamine detection with fine linearity, also showing good selectivity.

**Discussion:** The results from fetal bovine serum indicated the good applicability of the CDs in the determination of dopamine.

## 1 Introduction

Dopamine (DA) is a very important neurotransmitter, accounting for 80% of the catecholamine content in the mammalian brain (Wei et al., 2019; Costa and Schoenbaum, 2022). DA is mostly generated in adrenal glands and exists in the cationic form in living organisms, playing an essential role in almost all cognitive functions such as motor control, motivation, and learning, and its normal concentration is 10^−8^ to 10^−6^ M (Jiang et al., 2016; Zhu H et al., 2019). Studies have shown that abnormal DA concentrations cause a variety of complex mental diseases, for example, high DA concentrations can lead to schizophrenia, and lower DA concentrations can lead to Parkinson’s disease (Suzuki, 2017; Pan et al., 2022). Currently, known addictive drugs such as heroin, nicotine, and amphetamines could cause a rapid increase in DA levels in the body (Costa and Schoenbaum, 2022). In addition, the DA metabolism is mainly through two pathways, 3,4-dihydroxyphenylacetic acid and Homovanillic acid (HVA) approaches, and HVA concentration also affects the normal function of the organism (Jana et al., 2019). Due to these, it is urgently needed to construct an efficient, convenient, and accurate assay for DA concentration detection.

Up to now, multiple approaches have been devised to detect DA, including electrochemistry (Muti and Cantopcu, 2018; Asif et al., 2021), high-performance liquid chromatography (Syslova et al., 2011; Zhao et al., 2011), fluorescence methods [(Farahmand Nejad and Hormozi-Nezhad, 2017; Huang et al., 2020; Sivakumar et al., 2020)], and colorimetry (Zhu J L et al., 2019; Lai et al., 2021). Among them, electrochemistry and high-performance liquid chromatography are limited in their use due to complex operations and expensive equipment, while fluorescence detection of DA often requires the introduction of new fluorescent substances, which complicates the detection process (Wen et al., 2016; Wei et al., 2019; Hu et al., 2022). Colorimetry is of interest to researchers because of its simplicity, intuitiveness, sensitivity, and rapid response (Li J et al., 2023).

Carbon dots (CDs), as a kind of zero-dimensional carbon nanomaterials, have received considerable attention due to their advantageous characteristics, such as facile preparation, low cost, good water solubility and excellent optical properties (Huang et al., 2013). Therefore, there have been many reports refer to the detection of DA using CDs based on its fluorescence and enzyme properties (Huang et al., 2014; Naik et al., 2019; An et al., 2020; Zhou et al., 2023). The POD-like activity of nanomaterials allows catalytic oxidation of substrates such as TMB by H_2_O_2_, thus changing the absorbance of the system, which was widely used in the field of colorimetry detection (Ma et al., 2021; Li et al., 2022). For example, Lai et al. (2021) synthesized Pt600-GLP NCs with lower K_m TMB_ (0.17 mM) and K_m H2O2_ (2.06 mM) than that of the natural HRP, indicating that the Pt600-GLP NCs have a stronger affinity for the substrate. Since DA is reductive and could compete with TMB, the blue color of the system would be diminished, so Pt600-GLP NCs were used to catalyze the oxidation of TMB in the presence of H_2_O_2_ to analyze DA with a LOD of 0.66 μM (Lai et al., 2021). The reaction system of peroxidase-like nanozymes requires the addition of H_2_O_2_, which makes the system complex, and H_2_O_2_ is volatile, unstable, and easy to decompose over a long time. In contrast, oxidase-like nanozymes require simpler reaction conditions (avoiding the addition of H_2_O_2_). However, the oxidase-like nanozymes are easy to react with oxygen in air, and the activity is reduced, so it usually needs to be stored in an oxygen-free environment. Light-responsive oxidase-like nanozymes are only active under irradiation, and their activity is stable under light-avoidance conditions. Therefore, it is of great significance to develop light-responsive oxidase-like nanozymes with simple synthesis and high activity in the analysis field.

Carbon dots (CDs) have significant absorption in the visible light range and as well as enhanced electron transfer by doping, which has a very important potential in the field of light-responsive oxidase-like nanozymes (Saita and Kawasaki, 2023). The light-responsive catalytic activity of CDs has been employed for the detection of ions, L-ascorbic acid, and other substances based on the colorimetric method. For example, Sun et al. (2023) synthesized P-NCDs by hydrothermal method using triethylenetetramine hexaacetic acid as the precursor, which can convert oxygen in solution to singlet oxygen (^1^O_2_) under UV excitation at 365 nm, oxidizing TMB and gradually turning blue. Cysteine inhibited the catalytic activity of the P-NCDs, while its selective complexes with Hg^2+^ restored P-NCDs’ catalytic activity. The good linear range of detection at Hg^2+^ concentrations of 0.01–14 mM with a LOD of 3.1 nM (Sun et al., 2023). Li Y R et al. (2023) synthesized carbon dots by microwave-assisted method and the CDs can generate hydroxyl radicals to oxidize TMB under light irradiation. L-ascorbic acid, with a certain reducing property, inhibited the production of oxTMB by CDs after illumination, thus, achieving the detection of L-ascorbic with an LOD of 1.53 μM (Li Y R et al., 2023).

**FIGURE 1:**
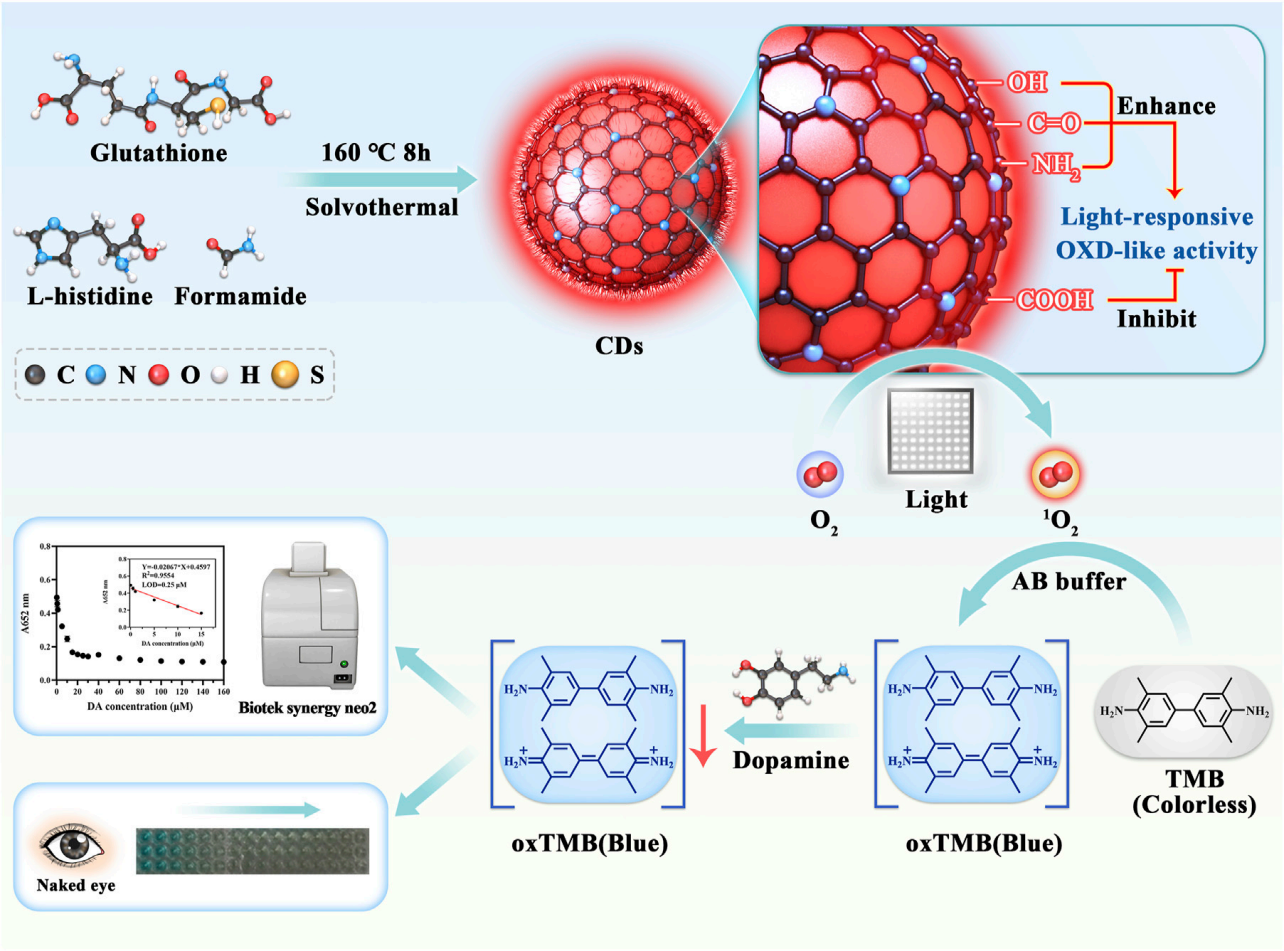
The illustration of the CDs preparation and colorimetric detection of DA.

Herein, as depicted in Figure 1, novel CDs were synthesized via the solvothermal route using glutathione, L-histidine, and formamide as sources for the detection of dopamine. Under LED irradiation, the CDs showed good oxidase-like activity and could catalyzed the oxidation of TMB to oxTMB. When dopamine (DA) was present in the CDs-coupled TMB system, it allowed the reduction of blue oxTMB to colorless TMB. Attributed to the changes of TMB absorption, the concentration of DA can be monitored quickly. Whar’s more, the results showed that the colorimetric method could successfully detect DA with a wide linear in the range of 0.5–15 μM with a LOD value of 0.25 μM. In addition, the CDs provide accurate detection of DA in fetal bovine serum, showing great potential for application. Then the surface functional groups (amino, carbonyl, hydroxyl, and carboxyl) of CDs were passivated separately using chemical modification methods to study the catalytic mechanism. We revealed that hydroxyl groups were the main site for the light-responsive catalytic activity of CDs, amino and carbonyl groups could enhance the catalytic activity, and the carboxyl groups had an inhibitory effect on the catalytic activity.

## 2 Experimental sections

### 2.1 Synthesis

Synthesis of CDs: 1.5 g of glutathione and 1.5 g of L-histidine were dissolved in 50 mL of formamide in a Teflon-lined autoclave. After heating the mixture at 160°C for 8 h and cooling it to room temperature, the reaction mixture was first dialyzed for 9 days and then further filtered through a 0.22 µm BIOSHARPP membrane filter. The CDs powder was obtained by freeze-drying the above solution.

#### 2.1.1 Synthesis of CDs-SPI

5 mg of CDs and 5 mg of 4-sulfophenyl isothiocyanate sodium salt monohydrate (SPI) were added to 7 mL of carbonation buffer (pH = 9). After reaction for 8 h at 4°C, the resulting mixture was dialyzed in ultrapure water for 4 days.

#### 2.1.2 Synthesis of CDs-PS

1 mL of CDs solution (5 mg/mL) and 1 mL of PS were added to 10 mL of 1, 4-dioxane. Then 1 mL triethylamine was added to the solution after 30 min. After stirring at 40°C for 24 h, the mixture was subjected to rotary evaporation to clear the organic solvent. The product obtained from the reaction was dissolved in deionized water. On the first day, it was dialyzed using NaCl solution (0.1 M). Then it was dialyzed with ultrapure water for 3 days.

#### 2.1.3 Synthesis of CDs-PS-hy

5 mg of CDs-PS was dissolved in 10 mL NaOH (0.5 M). After stirring at 40°C for 24 h, the mixture was neutralized with hydrochloric acid (0.1 M). The resulting mixture was dialyzed in ultrapure water for 4 days.

#### 2.1.4 Synthesis of CDs-NaBH_4_

Added 5 mg of CDs to 10 mL of NaBH_4_ solution (0.5 M), and the solution was stirred at 37°C for 24 h. The obtained solution was neutralized with hydrochloric acid (0.1 M). The next dialysis process was performed in ultrapure water (4 days).

### 2.2 Light-responsive oxidase-like activity of CDs

Solution A: 2.26 g of C_2_H_3_NaO_2_·3H_2_O (sodium acetate trihydrate), 0.175 g of C_6_H_8_O_7_·H_2_O (citric acid monohydrate); Solution B: 0.02 g of EDTA 2Na (ethylenediaminetetraacetic acid disodium salt dihydrate), 0.104 g of C_6_H_8_O_7_·H_2_O, they were both added to 50 mL of ultrapure water. CDs were dissolved in solution A, and TMB was dissolved in solution B. The experiments were divided into three groups as follows: 1) TMB + CDs; 2) Blank A + TMB; 3) Blank B + CDs, each group was set up with three replicate wells, TMB (1 mM) and CDs (10 μg/mL). The absorbance at 652 nm was measured using a Biotek Synergy Neo2 under an LED lamp (800 W) for 2, 5, 10, 20, and 30 min.

CDs were dissolved in solution A (final concentration 5 μg/mL) and TMB was dissolved in solution B (final concentrations 100, 200, 300, 400, and 500 μM, respectively). Kinetic measurements were performed using a UV-2700 spectrophotometer. The experimental groups added 400 μL of TMB and 400 μL of CDs solutions to a 1 cm cuvette, respectively, and absorbance at 652 nm was taken every 5 s of light (total 1 min), and the measurements were repeated three times. According to the above kinetic data, the Michaelis-Menten parameter was calculated:

$$V=V_{\max}\;\left[S\right]/\left(K_{m}+\left[S\right]\right)$$

Here V is the original velocity, and [S] is the concentration of TMB, V_max_ is the maximum reaction velocity and K_m_ is the Michaelis-Menten constant.

### 2.3 Detection of DA by CDs

CDs were dissolved in solution A (10 μg/mL, 100 μL), and TMB was dissolved in solution B (0.1, 0.2, and 0.4 mM, 100 μL). The volume of the total was 200 μL, and they were both mixed and shaken well. Different concentrations of DA solutions (final concentration 0–160 μM, 20 μL) were added after 5 min of illumination. After 5 min of reaction, the 652 nm absorption was measured with Biotek Synergy Neo2. The sample size for each group was 3. The assay was performed under the same conditions using 0.2 M pH4.0 acetate buffer saline. The LOD was calculated via the 3σ principle (*n* = 10). Pre-dilute the fetal bovine serum 20-fold and 100-fold with PBS, and dilute the DA solution with the above fetal bovine serum to 50, 100, and 150 μM. 100 μL of CDs dissolved in solution A and 100 μL of TMB dissolved in solution B were mixed with a final concentration of 10 μg/mL for CDs and 0.4 mM for TMB. After 5 min of light exposure, add 20 μL of DA solution and measure the absorption at 652 nm by a Biotek Synergy Neo2 after 5 min of reaction. The sample size for each group was 3.

## 3 Results and discussion

### 3.1 Characterization of CDs

**FIGURE 2:**
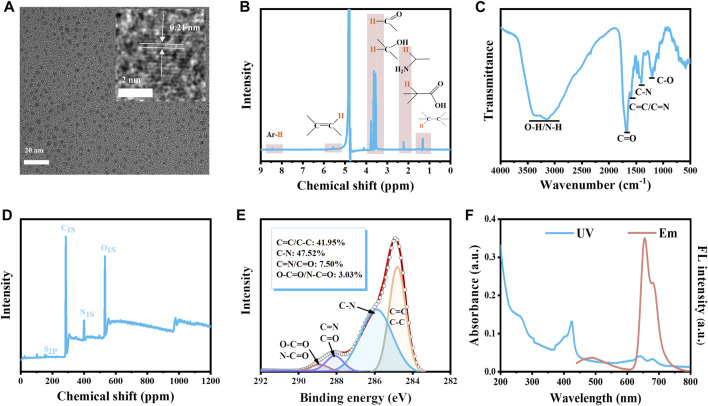
Characterization of CDs. **(A)** The TEM image of CDs. The inset is the HR-TEM image and lattice of the CDs. **(B)** ^1^H NMR spectrum **(C)** FT-IR spectrum **(D)** XPS results **(E)** High-resolution of C 1s of the CDs. **(F)** The UV spectrum and the fluorescence emission spectrum of CDs (10 μg/mL, ultrapure water).

The carbon dots (CDs) were obtained by hydrothermal method using glutathione, L-histidine, and formamide as raw materials. The morphology and structure of CDs were characterized by TEM, NMR, FT-IR, XPS, and Raman spectroscopy. From the TEM image (Figure 2A), it can be seen that the CDs exhibited good dispersibility. The particle size of the CDs was 3.42 ± 0.6 nm (Supplementary Figure S1). The measured crystalline spacing of CDs was 0.21 nm, which corresponded to the (100) crystalline surface of graphitic carbon. In the ^1^H-NMR spectrum of CDs (Figure 2B), the peaks in the region of 1.0–1.5 ppm represented the ^1^H of aliphatic hydrocarbons. The peak in the area of 1.8–2.5 ppm was attributed to α-H on the amino and carboxyl groups. The peak in the region of 3.5–3.8 ppm indicated the presence of α-H in the carbonyl and hydroxyl groups. In addition, two peaks at ∼5.5 and ∼8.5 ppm indicated the existence of olefins and aromatic rings, respectively. In the FT-IR spectrum of CDs, the broad absorption band of 3,000–3,600 cm^−1^ was caused by the O-H/N-H stretching vibration (Figure 2C). The peak at 1,684 cm^−1^ indicated the presence of the C=O stretching vibration (Figure 2C). The three peaks at 1,599 cm^−1^, 1,403 cm^−1,^ and 1,211 cm^−1^ were attributed to C=C/C=N, C-N, and C-O, respectively (Figure 2C). According to the XPS results of CDs, four peaks at 163.7, 285.0, 410.3, and 545.3 represented the S 2p, C 1s, N 1s, and O 1s, respectively. We also found that the CDs were composed of C, N, O, and S with molar ratios of 76.04%, 6.88%, 16.71%, and 0.38%, respectively (Figure 2D). The HR C 1s had been well fitted at 284.8, 285.9, 288.1, and 288.9 eV for the four peaks according to C=C/C-C (41.95%), C-N (47.52%), C=N/C=O (7.50%), and O-C=O/N-C=O (3.03%), respectively (Figure 2E). The HR N 1s had been well fitted at 399.9 and 400.6 eV according to pyrrolic N (70.14%) and amino (29.86%) (Supplementary Figure S2). The above results illustrated that CDs were covered by carboxyl, amino, hydroxyl, and carbonyl groups. The Raman spectrum of CDs exhibited two peaks at 1,350 cm^−1^ and 1,590 cm^−1^ belonging to the D and G bands, respectively (Supplementary Figure S3). The D band indicated the presence of defects and the G band indicated the presence of graphitic structure. The I(D)/I(G) ratio was 0.884 (Supplementary Figure S3), suggesting a large portion of defects existed in CDs. The CDs exhibited a narrow emission peak at 655 nm, together with a shoulder peak of 680 nm (Figure 2F). The CDs had multiple absorption bands in the wavelength range of 200–800 nm. The absorption band in the 200–300 nm could be explained by π→π* transition of the aromatic ring of CDs, and the absorption band at 350–450 nm could be explained by the π→π* transition of aromatic π systems including the functional groups such as C=N and C=O, while the absorption band at 600–750 nm was corresponding to the n→π* transition of the aromatic π systems, respectively (Pan et al., 2016). In addition, the CDs had a zeta potential of −14.07 ± 2.11 mV, indicating the stability of CDs under physiological conditions (Supplementary Figure S4).

### 3.2 Light-responsive OXD-like activity and catalytic mechanism of CDs

**FIGURE 3:**
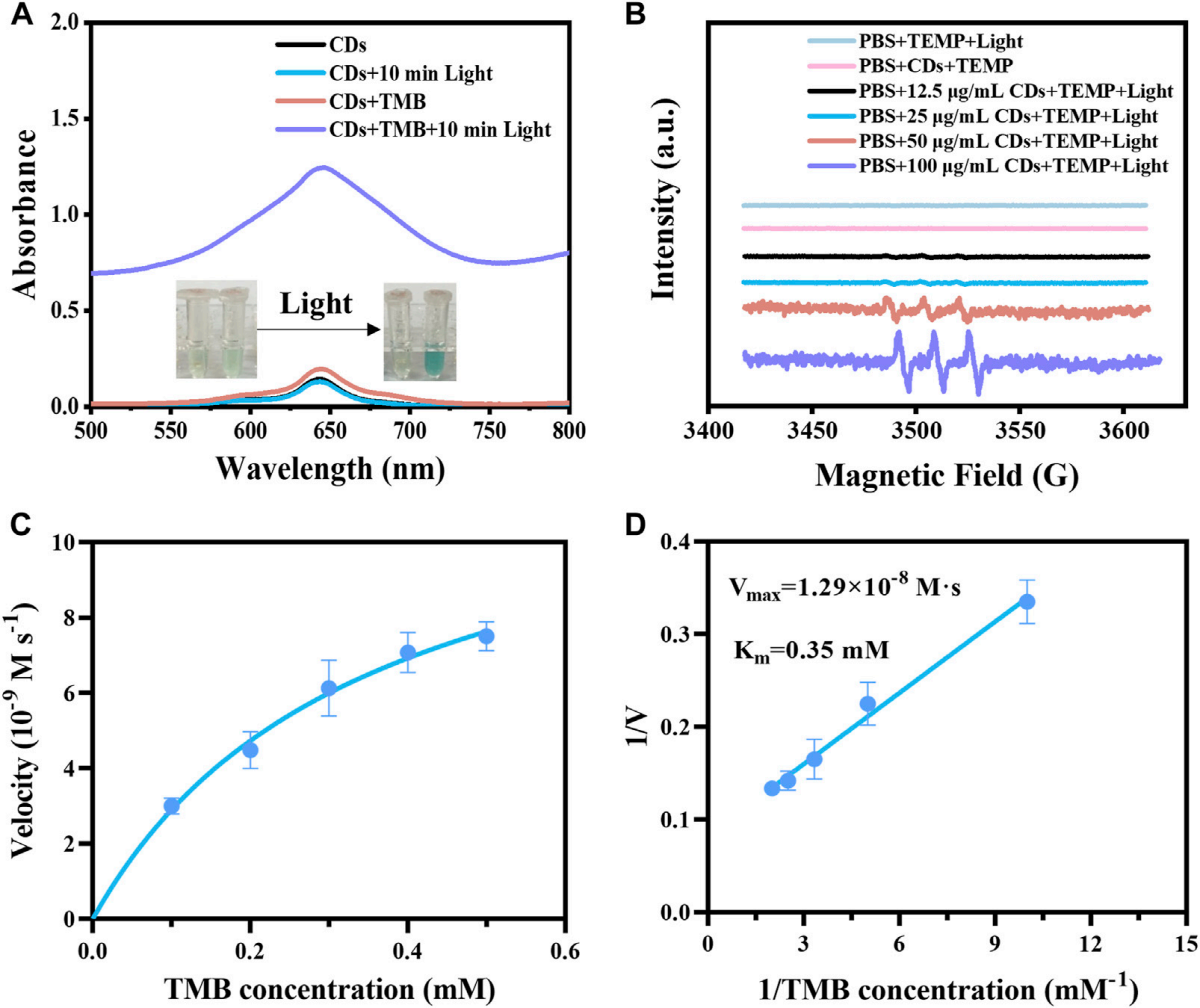
**(A)** UV–vis spectra of the samples, including CDs, CDs + Light, CDs + TMB, CDs + TMB + Light. Inset: photos of samples. **(B)** EPR spectra of different concentrations of CDs (LED lamp, 5 min of light stimulation) with the presence of TEMP. **(C)** Steady-state kinetic analysis of CDs was performed with TMB concentration as a variable. **(D)** Lineweaver-Burk plots of catalytic activity of CDs in the presence of TMB as a substrate.

We investigated the light-responsive OXD-like activity of CDs using TMB as a substrate. The CDs solution showed no significant change in the absorption peak at 652 nm after 10 min of irradiation. However, under the same light conditions, the color of the CDs and TMB mixture turned blue and the absorption peak red-shifted to 652 nm significantly enhanced. The above changes could be attributed to the oxidation of TMB by CDs under light stimulation, while the absorption of the CDs did not affect the characteristic absorption peak of TMB after oxidation (Figure 3A). The free radical types produced by CDs under irradiation were investigated by adding DMPO, TEMP, and DMSO to the mixture of CDs and TMB (Liang et al., 2020). DMPO can capture superoxide anion and hydroxyl radical, TEMP can capture singlet oxygen, and DMSO can capture hydroxyl radical. When the generated radicals were captured, the content of oxTMB decreased, as well the characteristic absorption at 652 nm decreased. Therefore, the type of generated radicals was determined by detecting the absorption value at 652 nm. When 25 mM TEMP was added to the mixture of the CDs and TMB, the absorption at 652 nm was significantly reduced after illumination for 1 min and 5 min, which was more obvious when the TEMP concentration was 50 mM. While the absorption at 652 nm did not change significantly after the addition of DMPO and DMSO under the same irradiation condition (Supplementary Figure S5), which indicated that the radical generated by CDs under irradiation was singlet oxygen. The type of generated free radical was also analyzed by EPR spectroscopy. We observed a 1:1:1 characterized singlet oxygen three-line spectrum in the CDs solution after 5 min of light stimulation, and the intensity of the spectrum was enhanced with the increase of CDs concentrations (Figure 3B). The above results illustrated that CDs generated singlet oxygen under irradiation. Based on the enzyme kinetic theory, the catalytic activity of CDs was further studied with TMB as a substrate. The Michaelis-Menten and Lineweaver-Burk double-reciprocal curves were gained by the steady-state kinetic method (Figures 3C, D). The V_max_ and K_m_ values of the CDs with the substrate TMB were displayed at 1.29 × 10^−8^ M s^−1^ and 0.35 mM, respectively (Figure 3D). CDs showed comparable V_max_ values, and lower K_m_ values, even when compared to single-atom oxidase-like nanozyme and HRP (Supplementary Table S1). The lower K_m_ indicates the CDs as light-responsive oxidase mimics are more affinitive to TMB.

**FIGURE 4:**
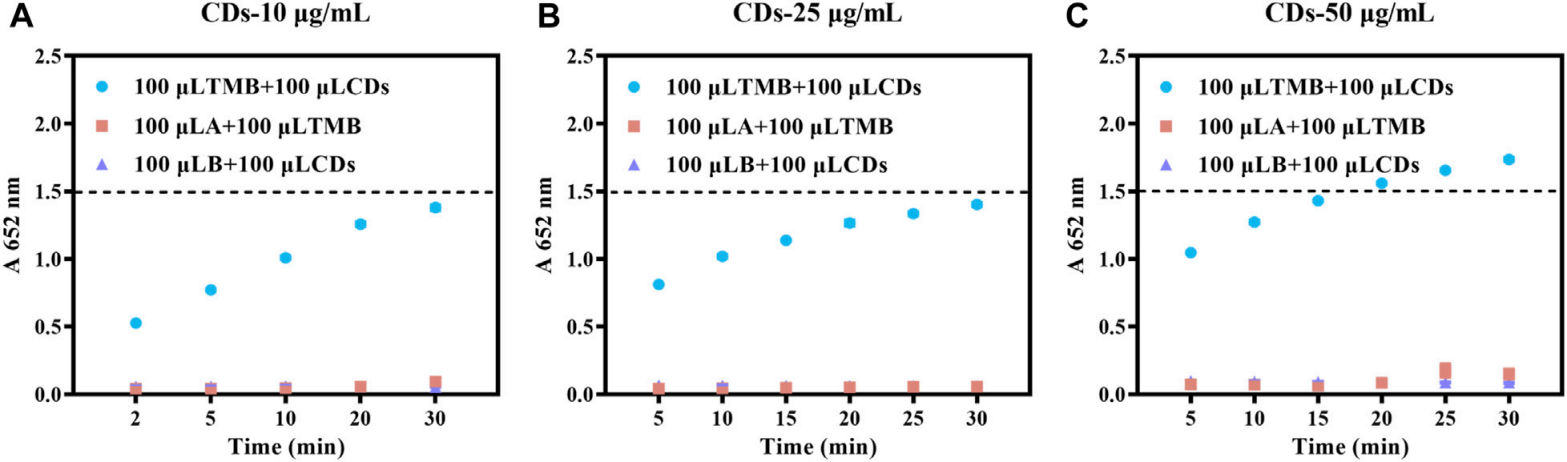
Effect of CDs concentration on the catalytic activity of CDs (AB buffer, TMB:1 mM). **(A)** 10 **(B)** 25 **(C)** 50 μg/mL.

We next investigated the effects of CDs concentration, irradiation time, and pH on the light-responsive catalytic activity of CDs. The absorbance value at 652 nm of oxTMB was 1.38, 1.40, and 1.74 for CDs at 10, 25, and 50 μg/mL after 30 min of illumination (Figures 4A–C). Compared with the light-responsive catalytic activity of CDs at 10 μg/mL, the catalytic activity of CDs at 25 and 50 μg/mL showed an increase, indicating that the catalytic activity of CDs was concentration-dependent and its catalytic activity increased with the incremental concentration. It was worth mentioning that the catalytic activity of CDs increased significantly with the increase in irradiation time (Figure 4). The light-responsive catalytic activity of CDs at different pH (3.6, 4, 4.5, 5, 5.5, and 5.8) were presented in Supplementary Figure S6. The absorption value at 652 nm reached maximum value when the pH was 4.0, and the absorption value of oxTMB at 652 nm was more than 2 after 20 min of irradiation (Supplementary Figure S6B). In addition, we prepared several similar CDs by replacing L-histidine with L-arginine, L (+)-glutamic acid, L (+)-cysteine, or L-aspartic acid, producing Arg-CDs, Glu-CDs, Cys-CDs, and Asp-CDs, respectively. All of them displayed light-responsive catalytic activity (Supplementary Figure S7). However, the absorption of oxTMB at 652 nm of Arg-CDs (1.12), Glu-CDs (0.70), Cys-CDs (0.49), and Asp-CDs (0.85) were lower than that of the His-CDs (1.38) after 30 min of irradiation, indicating that raw materials determine the catalytic activity of CDs.

### 3.3 Effect of surface functional groups on light-responsive oxidase-like activity

**FIGURE 5:**
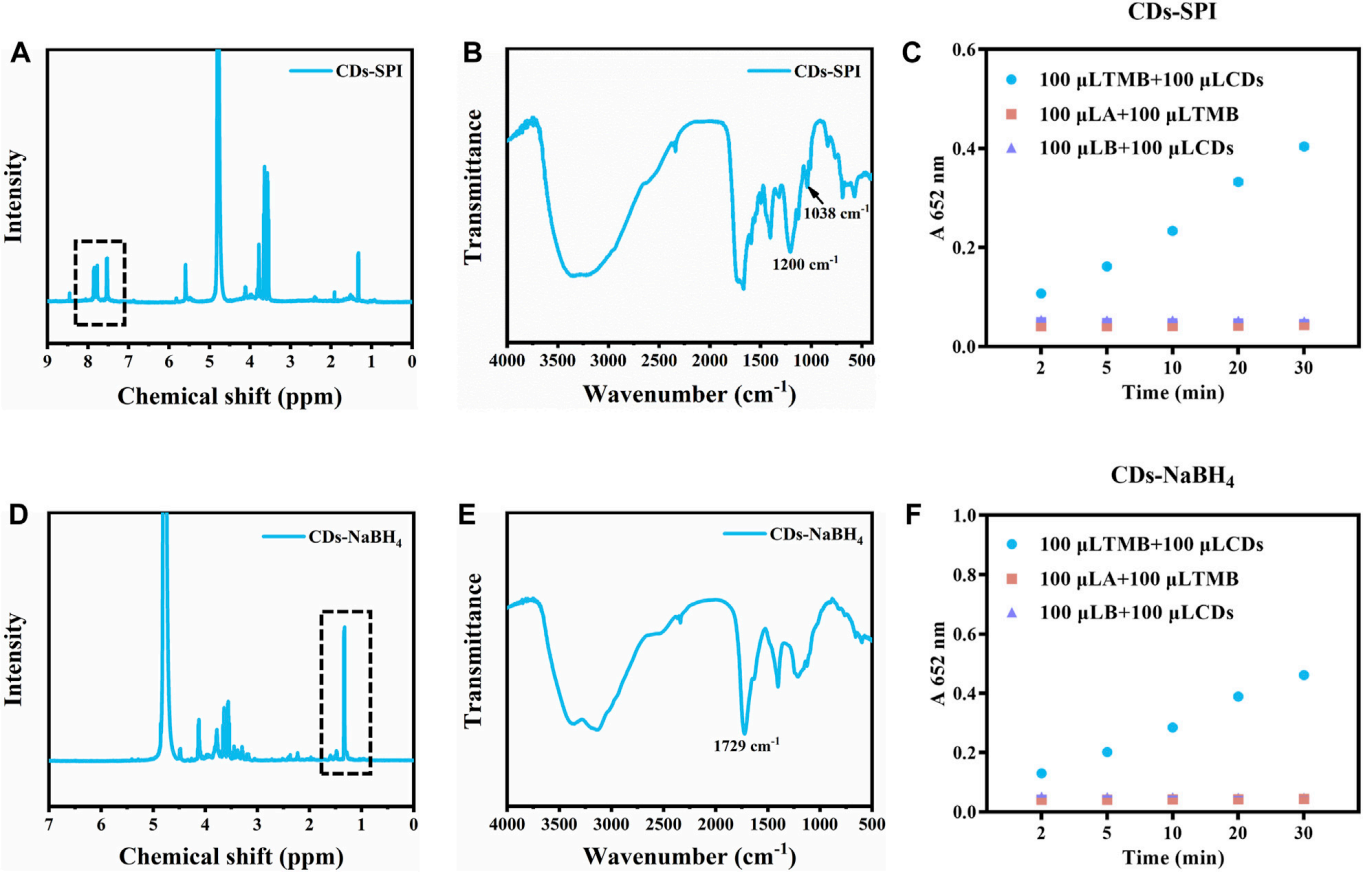
**(A)** ^1^H NMR spectrum of CDs-SPI. **(B)** FT-IR spectrum of CDs-SPI. **(C)** The catalytic activity of CDs-SPI. **(D)** ^1^H NMR spectrum of CDs-NaBH_4_. **(E)** FT-IR spectrum of CDs-NaBH_4_. **(F)** The catalytic activity of CDs-NaBH_4_ (10 μg/mL, AB buffer, TMB:1 mM).

A variety of surface modifications were performed to explore and uncover the structure-property and catalytic activity sources of CDs. Firstly, the amino groups were reacted with 4-sulfophenyl isothiocyanate sodium salt (SPI) to study whether the substitution reaction of amino groups affected the light-responsive catalytic activity of CDs. As shown in ^1^H-NMR of CDs-SPI (Figure 5A), the peaks in the range of 7.0–8.2 ppm corresponding to ^1^H in the benzene ring of SPI appeared, indicating that the amino groups of CDs successfully reacted with SPI. As shown in the FT-IR spectrum of CDs-SPI (Figure 5B), the peak appeared at 1,038 cm^−1^ indicated the presence of sulphonic acid sodium salt. In addition, the peaks located at 580 to 900 cm^−1^ indicate the enhancement of out-of-plane bending vibration of the aromatic nucleus. We found that the light-responsive catalytic activity of CDs-SPI was significantly decreased compared with CDs, and the absorption of oxTMB at 652 nm after 30 min of irradiation was only 0.404 (Figure 5C), which demonstrated that amino groups can enhance the catalytic activity of CDs.

Sodium borohydride is a common reductant that can reduce carbonyl. As demonstrated in ^1^H-NMR of CDs reduced by NaBH_4_ (CDs-NaBH_4_), a significant increase of the peaks in the region of 1.0–2.0 ppm was observed, indicating that the C=O of CDs was reduced to C-O, respectively (Figure 5D). Meanwhile, in the FT-IR spectrum of CDs-NaBH_4_ (Figure 5E), the characteristic peak of C=O at 1729 cm^−1^ decreased significantly, confirming the successful passivation of C=O. The light-responsive catalytic activity of CDs-NaBH_4_ was also significantly decreased, and the absorption of oxTMB at 652 nm after 30 min of irradiation was 0.461, which was slightly stronger than CDs-SPI (0.404) (Figure 5F), which demonstrated that carbonyl groups can also enhance the catalytic activity of CDs.

**FIGURE 6:**
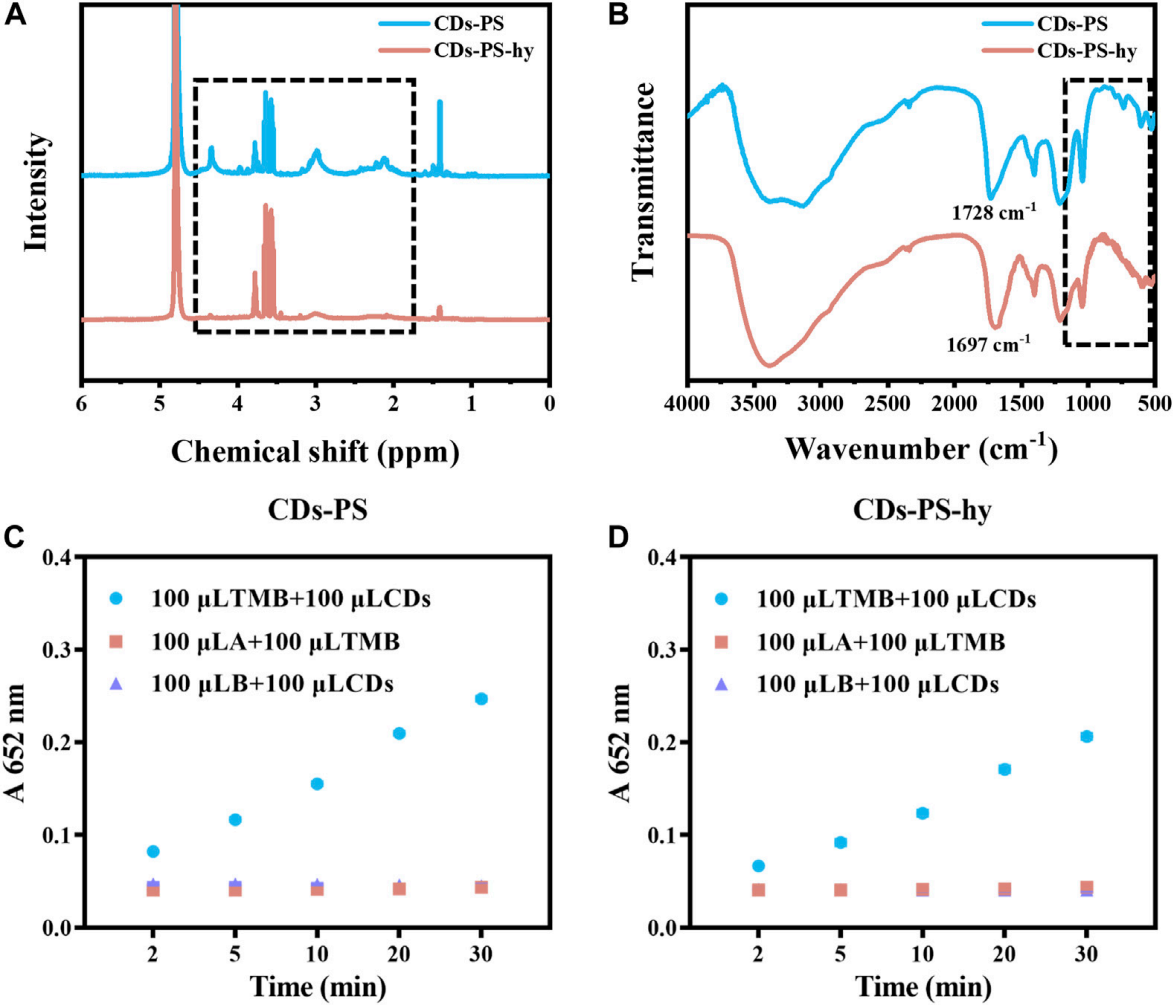
**(A)** ^1^H NMR spectrum of CDs-PS and CDs-PS-hy. **(B)** FT-IR spectrum of CDs-PS and CDs-PS-hy. **(C)** The catalytic activity of CDs-PS (10 μg/mL, AB buffer, TMB:1 mM). **(D)** The catalytic activity of CDs-PS-hy (10 μg/mL, AB buffer, TMB:1 mM).

We used 1,3-propane sulfonate (PS) to passivate the carboxyl and hydroxyl groups of the CDs surface (Gao et al., 2023; Liu et al., 2023). The carboxyl and hydroxyl react with PS to produce ester and ether, respectively, while the ester is easily hydrolyzed under alkaline conditions. By hydrolyzing the CDs-PS with NaOH, CDs with the hydroxyl groups passivated could be obtained. After being modified by PS, the new peaks at 2.1, 3.0, and 4.3 ppm appeared in the ^1^H-NMR of CDs-PS, which corresponded to the ^1^H of β, α, γ of -SO_3_^−^ and the above three peaks in CDs-PS-hy were significantly reduced (Figure 6A). In the FT-IR spectrum of CDs-PS (Figure 6B), the stretching vibration of C=O located at 1,684 cm^−1^ shifted to 1728 cm^−1^, suggesting the generation of ester. The stretching vibration of C=O in CDs-PS-hy was back to 1,697 cm^−1^, which demonstrates the recovery of carboxyl groups. Meanwhile, the characteristic absorption peaks of sulfonic acid groups appeared at 528, 610, and 1,045 cm^−1^ in the FT-IR spectra of CDs-PS while they decreased in the spectra of CDs-PS-hy (Figure 6B). After the carboxyl and hydroxyl groups on the surface of CDs were passivated simultaneously, the light-responsive catalytic activity of CDs-PS decreased sharply, and the absorption of oxTMB at 652 nm after 30 min of irradiation was 0.247, which was slightly lower than CDs-SPI and CDs-NaBH_4_ (Figure 6C). After hydrolysis under NaOH solution, the carboxyl group was released. The light-responsive catalytic activity of CDs-PS-hy was further decreased after the carboxyl group had been released, and the absorption of oxTMB after 30 min of irradiation was 0.206 (Figure 6D). This demonstrated that the hydroxyl groups can greatly increase the light-responsive catalytic activity of CDs, while the slight amount of carboxyl groups acts as an inhibitor of catalytic activity.

### 3.4 Colorimetric detection of DA

**FIGURE 7:**
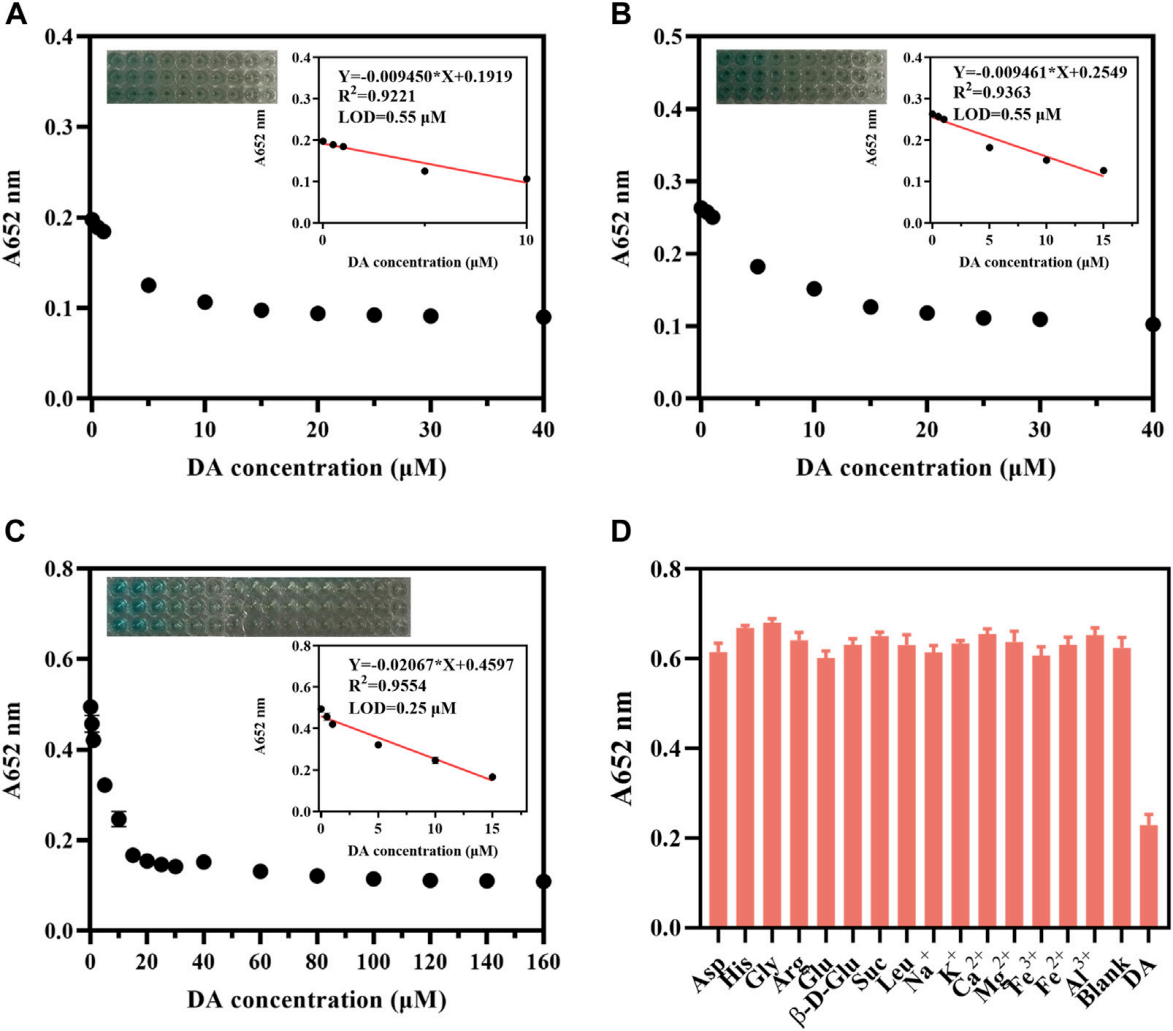
CDs detection DA (10 μg/mL, AB buffer). The change in absorbance at 652 nm of CDs (10 μg/mL, AB buffer) + **(A)** TMB (0.1 mM, AB buffer); **(B)** TMB (0.2 mM, AB buffer); **(C)** TMB (0.4 mM, AB buffer) upon addition of different concentrations of DA. **(D)** Selectivity of CDs (10 μg/mL, AB buffer)–coupled TMB (0.4 mM, AB buffer) for detecting DA in the presence of various metal ions, sugars, and amino acids (All the substance concentrations were 100 μM).

DA is a substance with reducing properties and plays an essential role in living organisms, so we tried to detect it using CDs and TMB. The catalytic oxidation of TMB by CDs under illumination can be inhibited by substances with reducing properties. Therefore, we detected DA by the CDs-coupled TMB system (AB buffer) to achieve a better LOD and linear detection range of DA. The absorption of the CDs-coupled with the TMB system at 652 nm decreased significantly with increasing DA concentration, the rate of decrease was changed at different TMB concentrations. And the response of the CDs coupled with the TMB system to DA was most sensitive at 0.4 mM TMB (Figures 7A–C). When the TMB concentration was 0.4 mM, the absorption at 652 nm was linearly related to DA concentration in the range of 0.5–15 μM, and the LOD was 0.25 μM (Figure 7C). Similarly, we detected DA with the CDs-coupled TMB system (replacing AB buffer with 0.2 M pH 4.0 acetate buffer saline). With the increased concentration of DA, the absorption at 652 nm of the CDs-coupled TMB system was also significantly decreased, with a good rate of decreasing at 0.2 mM TMB at this time, the absorption at 652 nm was linearly related to DA concentration in the range of 5–60 μM, and the LOD was 3.04 μM (Supplementary Figure S8). In summary, we used AB buffer with TMB of 0.4 mM for DA detection, where the LOD reached 0.25 μM.

The selectivity of the CDs-coupled TMB system (AB buffer) towards DA was investigated. We chose common metal ions such as Na^+^, K^+^, Ca^2+^, Mg^2+^, Fe^3+^, Fe^2+^, Al^3+^, β-D-glucopyranose, sucrose, and amino acids containing aspartic acid, histidine, glycine, arginine, glutamic acid, and leucine to evaluate the selectivity. All the above substances showed hardly a significant interfering role in the DA assay, indicating that this CDs-coupled TMB colorimetric method has excellent selectivity (Figure 7D). This CDs-coupled TMB-based DA assay has a more sensitive LOD and simple reaction system when compared to other systems (Supplementary Table S2).

### 3.5 Detection of DA in serum samples

We next evaluated the ability of the CDs-coupled TMB system (AB buffer) to detect DA in complex environments. When the serum was diluted 200-fold, the acceptable recovery rate ranged from 90.75% to 133.56% with the RSD values being less than 4% (Table 1). When the serum was diluted 1000-fold, the acceptable recovery rate ranged from 90.43% to 147.75%, with RSD values being less than 5% (Table 1). These results indicated that the system can be used to detect DA in real samples.

**TABLE 1:** CDs-coupled TMB system (AB buffer) detection of DA in diluted serum (*n* = 3).

	Sample	Added amount (μM)	Measured (μM)	Recovery (%)	RSD% (*n* = 3)
Diluted 200-fold	Serum	5	6.68	133.56	1.90
	Serum	10	10.98	109.84	3.55
	Serum	15	13.61	90.75	1.31
Diluted 1000-fold	Serum	5	7.39	147.75	4.57
	Serum	10	11.31	113.06	2.10
	Serum	15	13.56	90.43	1.76

## 4 Conclusion

In general, The CDs with light-responsive catalytic activity were prepared by hydrothermal method using glutathione, L-histidine, and formamide as sources. According to the competitive effects, a colorimetric assay for DA detection based on a CDs-coupled TMB system was fabricated and achieved a low LOD of 0.25 μM and a broad linear range of 0.5–15 μM with good selectivity. Through surface modifications, we revealed that the amino, carbonyl, carboxyl, and hydroxyl on the surface of CDs affect its catalytic activity. Hydroxyl, amino, and carbonyl groups can enhance the light-responsive catalytic activity of CDs, and the carboxyl groups have an inhibiting effect on their catalytic activity. Furthermore, the CDs-coupled TMB system can be employed for detecting the DA in fetal bovine serum. This work identifies the light-responsive catalytic activity mechanism of CDs and realizes the accurate DA assay in real samples.

## Data Availability

The original contributions presented in the study are included in the article/Supplementary Material, further inquiries can be directed to the corresponding authors.

## Abbreviations

DA, Dopamine; CDs, Carbon dots; ^1^O_2_, Singlet oxygen; TMB, 3,3′,5,5′-tetramethylbenzidine dihydrochloride; LOD, Limit of detection; HVA, Homovanillic acid; POD, Peroxidase; OXD, Oxidase; Pt600-GLP NCs, Ganoderma lucidum polysaccharide stabilized platinum nanoclusters; P-NCDs, Nitrogen-doped carbon quantum dots; HRP, Horseradish peroxidase; SPI, 4-sulfophenyl isothiocyanate sodium salt monohydrate; PS, 1,3-propane sultone; TEM, Transmission electron microscopy; XPS, X-ray photoelectron spectroscopy; NMR, Nuclear magnetic resonance spectroscopy; FT-IR, Fourier infrared spectrometer; HR, High-resolution; DMPO, 5,5-dimethyl-1-pyrroline N-oxide; TEMP, 4-amino-2,2,6,6-tetramethylpiperidine; EPR, Electron paramagnetic resonance; UV-Vis, Ultraviolet and visible spectrophotometry.
