# Supplementary material for: Carbon dots with light-responsive oxidase-like activity for colorimetric detection of dopamine and the catalytic mechanism
Source: Front Chem. 2023 Oct 12;11:1288418. doi: 10.3389/fchem.2023.1288418 (PMC10601655; doi:10.3389/fchem.2023.1288418)
Supplement: Supplementary file 1 [file DataSheet1.DOCX]

**Supporting Information**

**Experimental Procedures**

**Reagents and materials**

L-histidine, glutathione (reduced), formamide, 1,3-propanesulfonate (PS), magnesium chloride (MgCl_2_), L-aspartic acid, L-arginine, L(+)-cysteine, glycine, 4-amino-2,2,6,6-tetramethylpiperidine (TEMP), dopamine hydrochloride, L(+)-glutamic acid, 5,5-dimethyl-1-pyrroline N-oxide (DMPO) and sucrose were purchased from Aladdin Chemical Reagent Co., Ltd (Shanghai, China). 1, 4-dioxane, calcium chloride (CaCl_2_), sodium acetate trihydrate (C_2_H_3_NaO_2_·3H_2_O), citric acid monohydrate (C_6_H_8_O_7_·H_2_O), potassium chloride (KCl), and sodium borohydride (NaBH_4_) were purchased from Sinopharm Chemical Reagent Co., Ltd (Shanghai, China). 4-Sulfophenyl isothiocyanate sodium salt monohydrate was purchased from Sigma-Aldrich Co., Ltd (Shanghai, China). Sodium chloride (NaCl), sodium hydroxide (NaOH), and sodium hydrogen carbonate (NaHCO_3_) were purchased from Tianli Enterprise Group Co., Ltd (Tianjin, China). β-D-glucopyranose and 3,3′,5,5′-tetramethylbenzidine dihydrochloride (TMB) were purchased from Shanghai Macklin Biochemical Co., Ltd (Shanghai, China). Triethylamine (TEA) was purchased from Energy Chemical Co., Ltd (Shanghai, China). L-leucine was purchased from Shanghai Lanji technology development Co., Ltd (Shanghai, China). Ethylenediaminetetraacetic acid disodium salt dihydrate (EDTA Na2) was purchased from Bo Far Tyrone Biological Technology Co., Ltd (Beijing, China). Fetal bovine serum was purchased from ExCell Bio Co., Ltd (Shanghai, China). Acetic acid glacial was purchased from T·J Kemao Chemical Reagent Co., Ltd (Tianjin, China). Hydrochloric acid (HCl), and nitric acid (HNO_3_, 65%~68%) were purchased from local supplies. All aqueous solutions were prepared with deionized water (18.2 MΩ·cm, Millipore).

**Instruments**

Thermo Fisher Nicolet 5700 and Thermo Fisher DXR2xi were used to measure FTIR spectra and Roman spectra, respectively. X-ray photoelectron spectroscopy was obtained by a Thermo Fisher ESCALAB Xi^+^. SHIMADZU UV-2700 Spectrophotometer was used to measure UV-Vis absorption spectra. An AVANCE III HD spectrometer (600 MHz) was employed to perform the proton magnetic resonance (^1^H NMR) spectra. A Malvern Zetasizer Nano ZSE was used to record the Zeta potential. Transmission electron microscopy (TEM) and high-resolution TEM images were obtained from a JEM-2100 microscope. The fluorescence spectra were obtained from a HITACHI-F4700. Biotek Synergy Neo2 was used to measure the OD value at 652 nm. Electron paramagnetic resonance (EPR) spectra were obtained by a Bruker A300-9.5/12 at room temperature.

**Supplementary Figures**

**
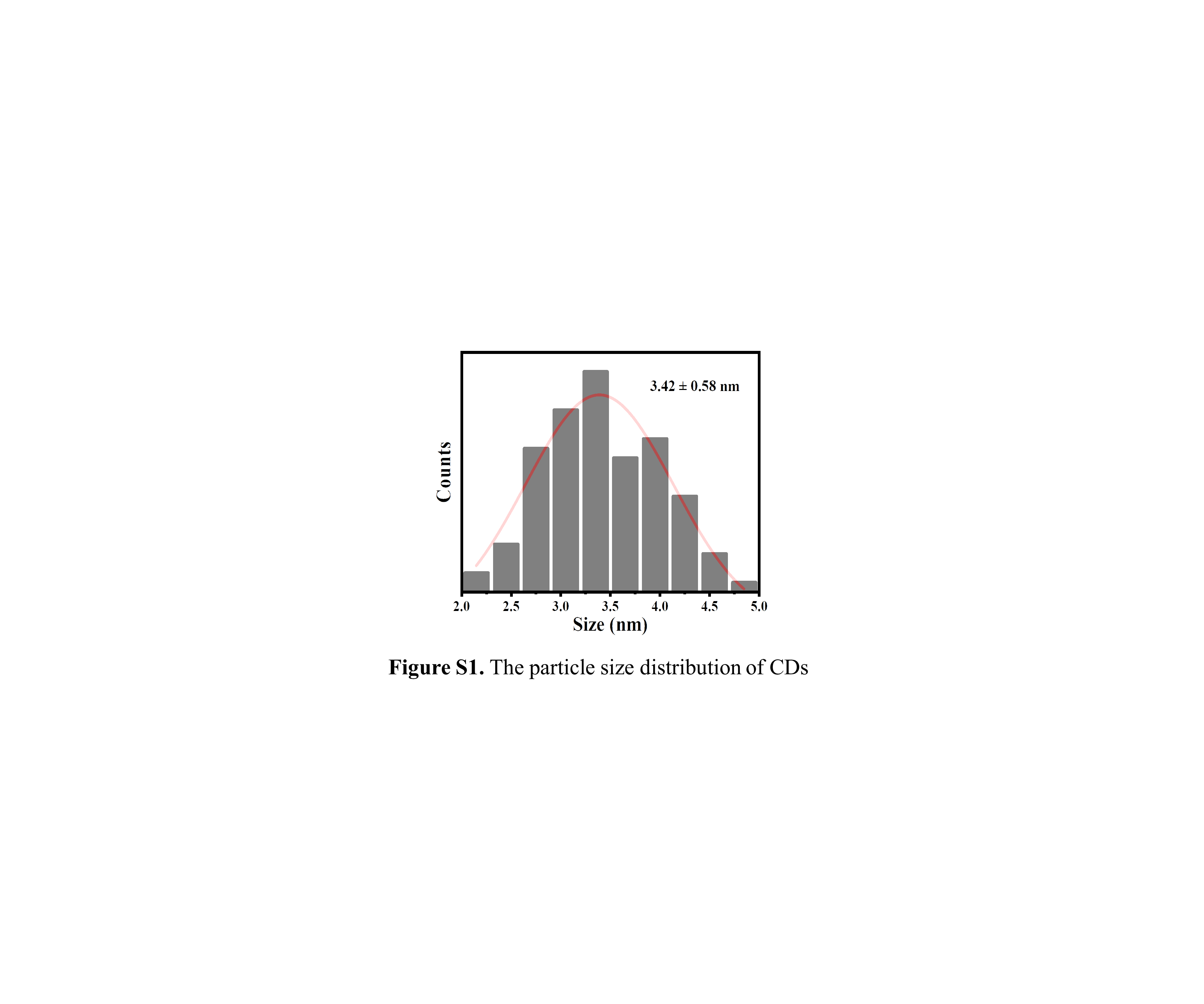
**

**Figure S1.** The particle size distribution of CDs.

**
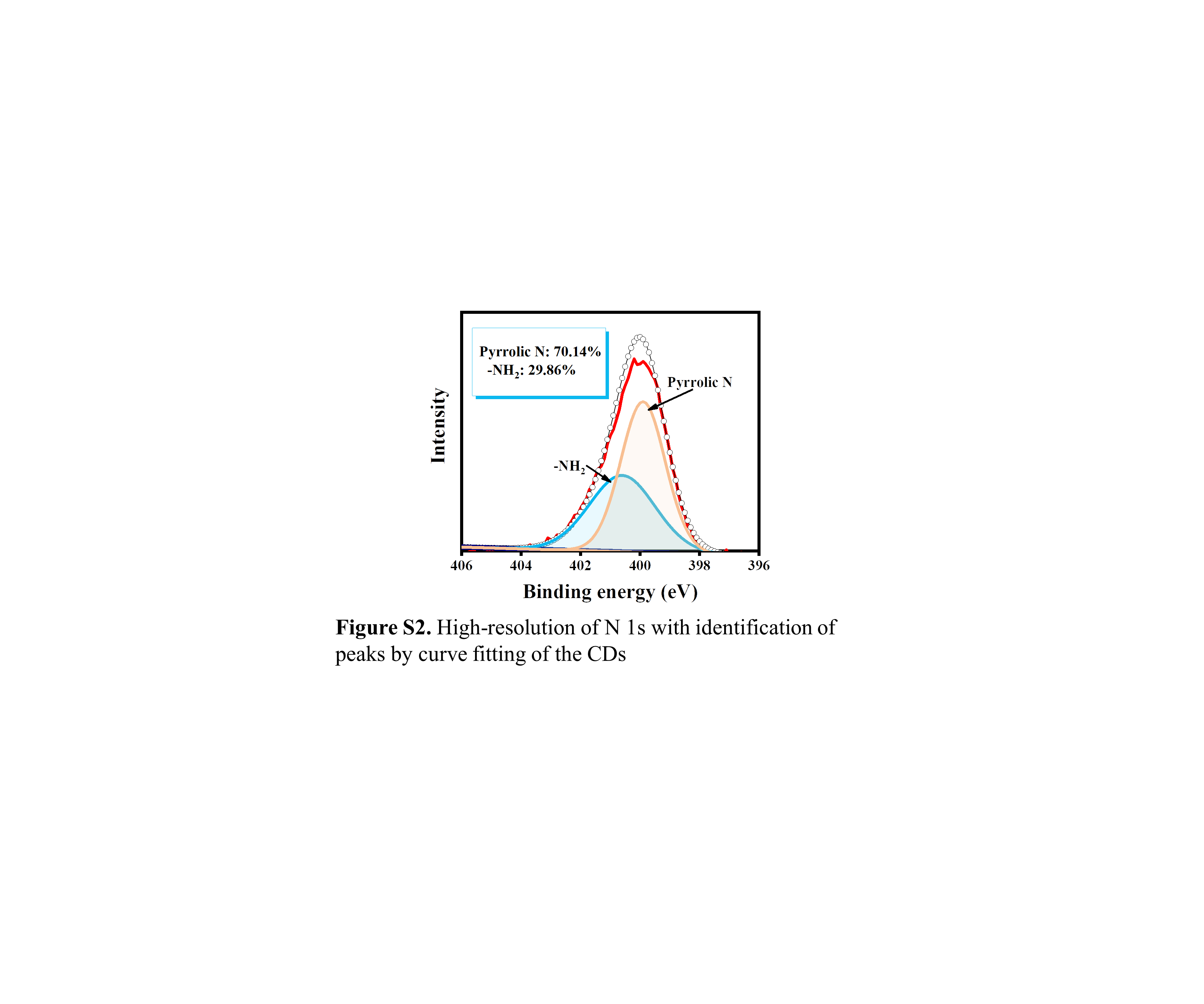
**

**Figure S2.** High-resolution of N 1s with identification of peaks by curve fitting of the CDs.


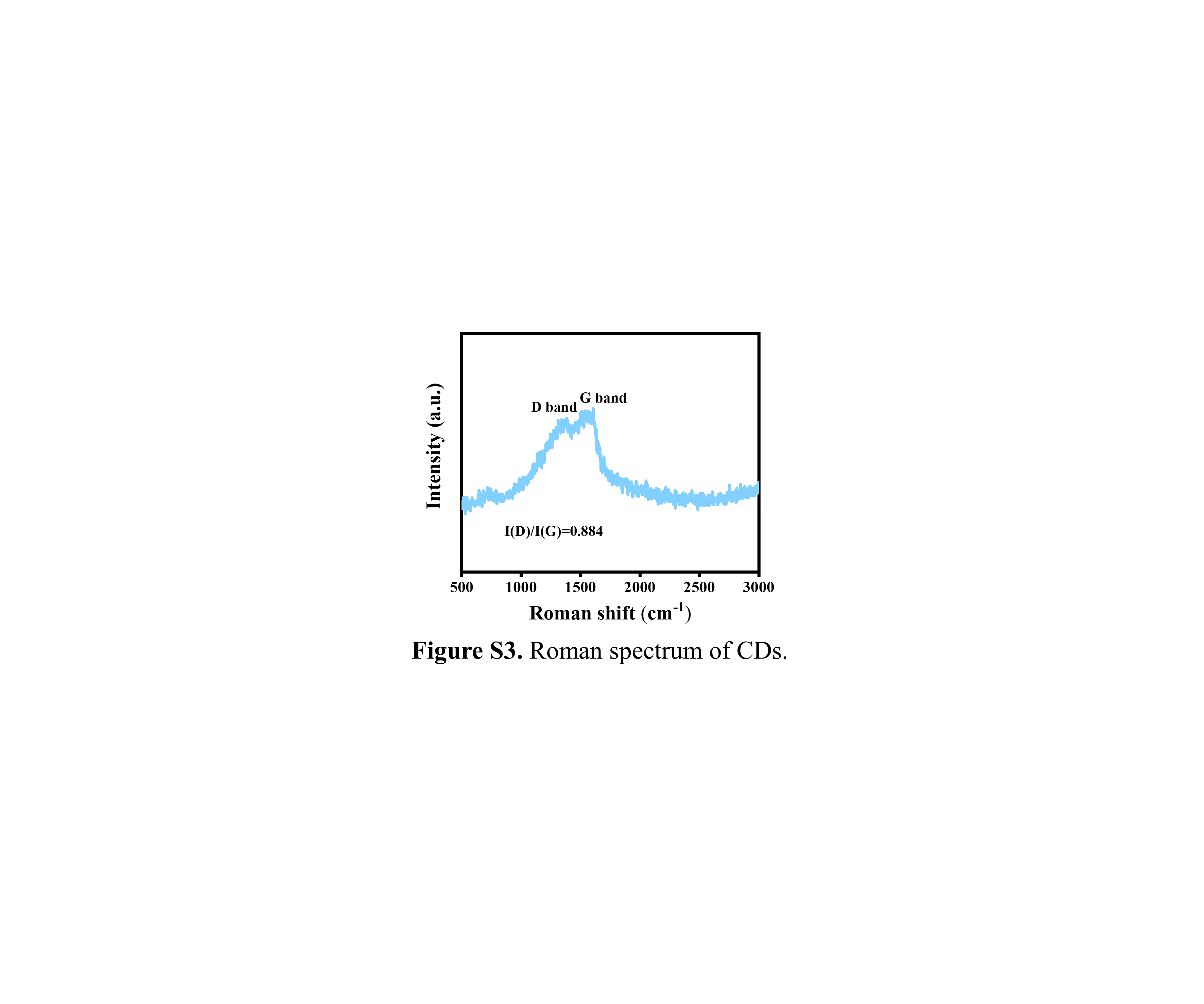


**Figure S3.** The Roman spectrum of CDs.

**
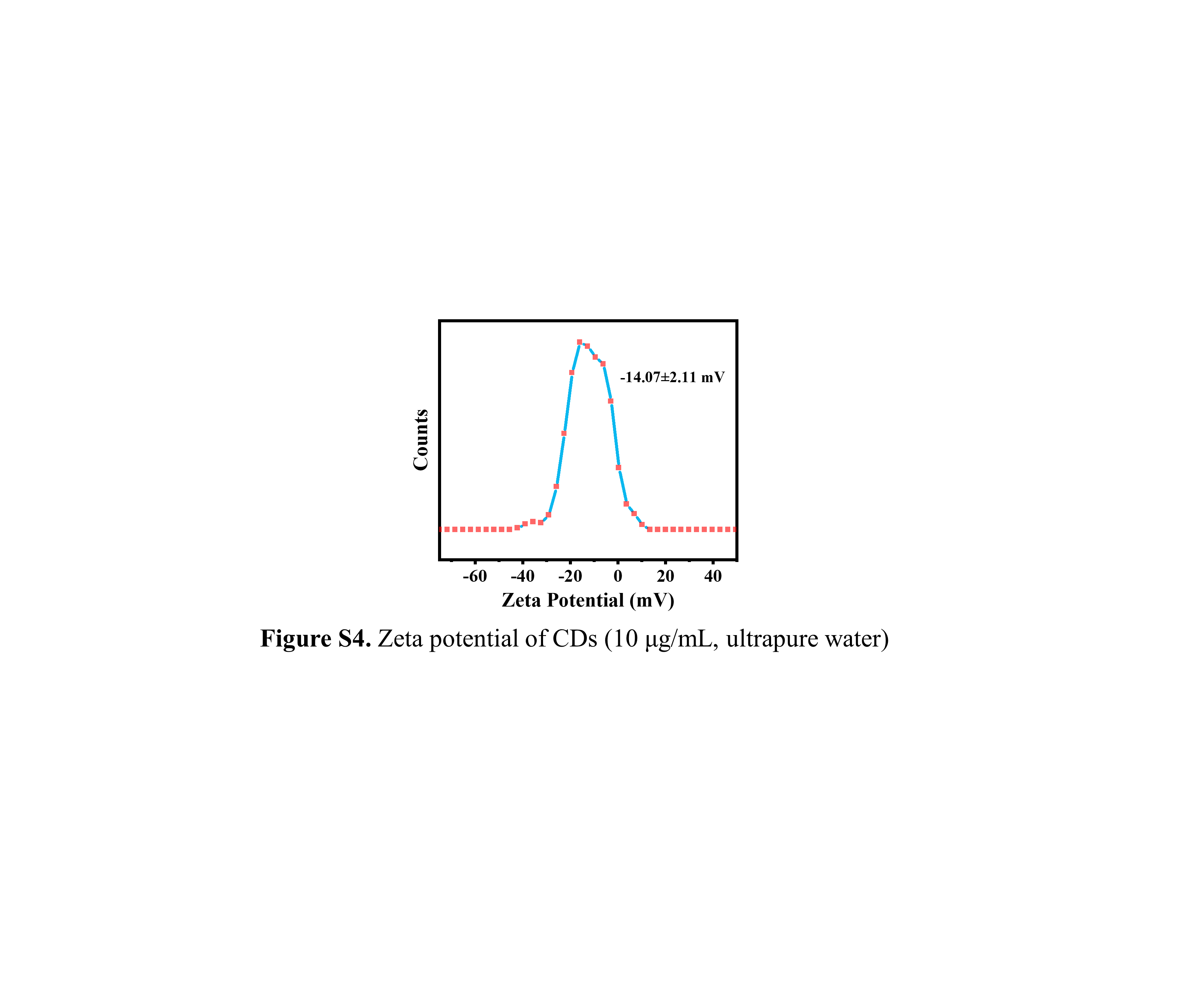
**

**Figure S4.** The Zeta potential of CDs (10 μg/mL, ultrapure water).


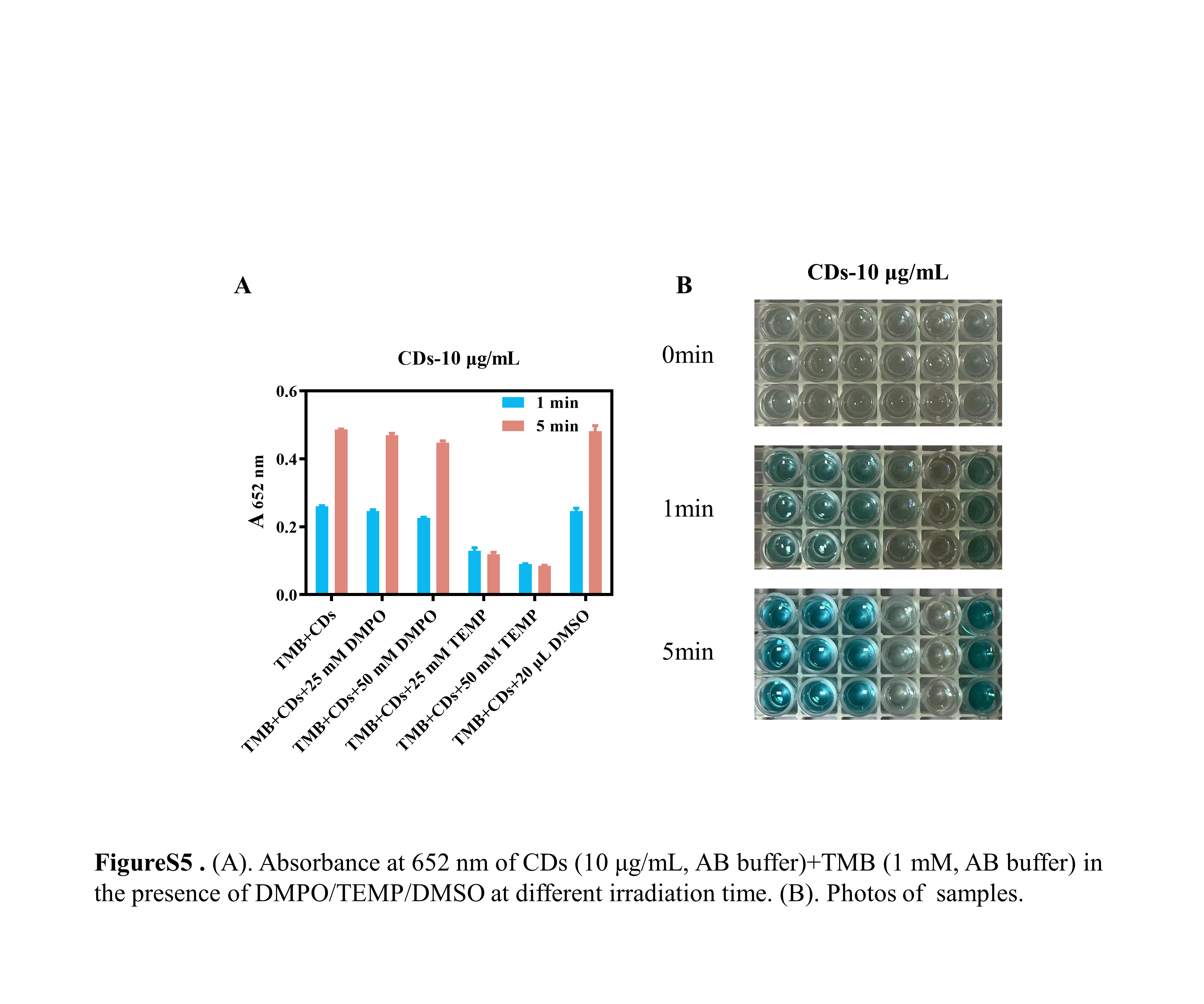


**Figure S5. (A)** Absorbance at 652 nm of CDs (10 μg/mL, AB buffer) + TMB (1 mM, AB buffer) in the presence of DMPO/TEMP/DMSO at different irradiation times. **(B)** Photos of samples.


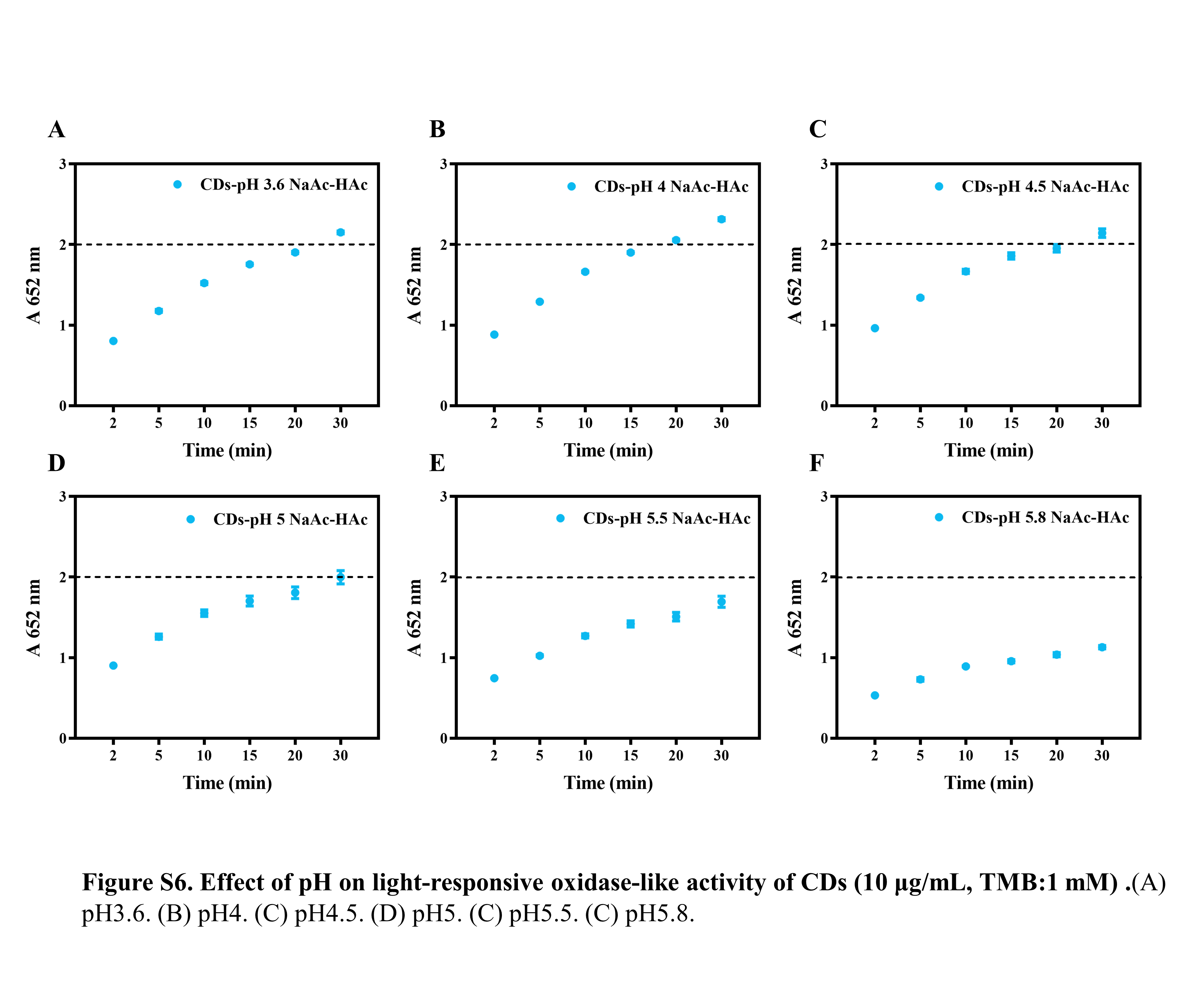


**Figure S6.** Effect of pH on catalytic activity of CDs (10 μg/mL, TMB:1 mM, 0.2 M pH4.0 acetate buffer saline). **(A)** pH3.6 **(B)** pH4 **(C)** pH4.5 **(D)** pH5 **(E)** pH5.5 **(F)** pH5.8.


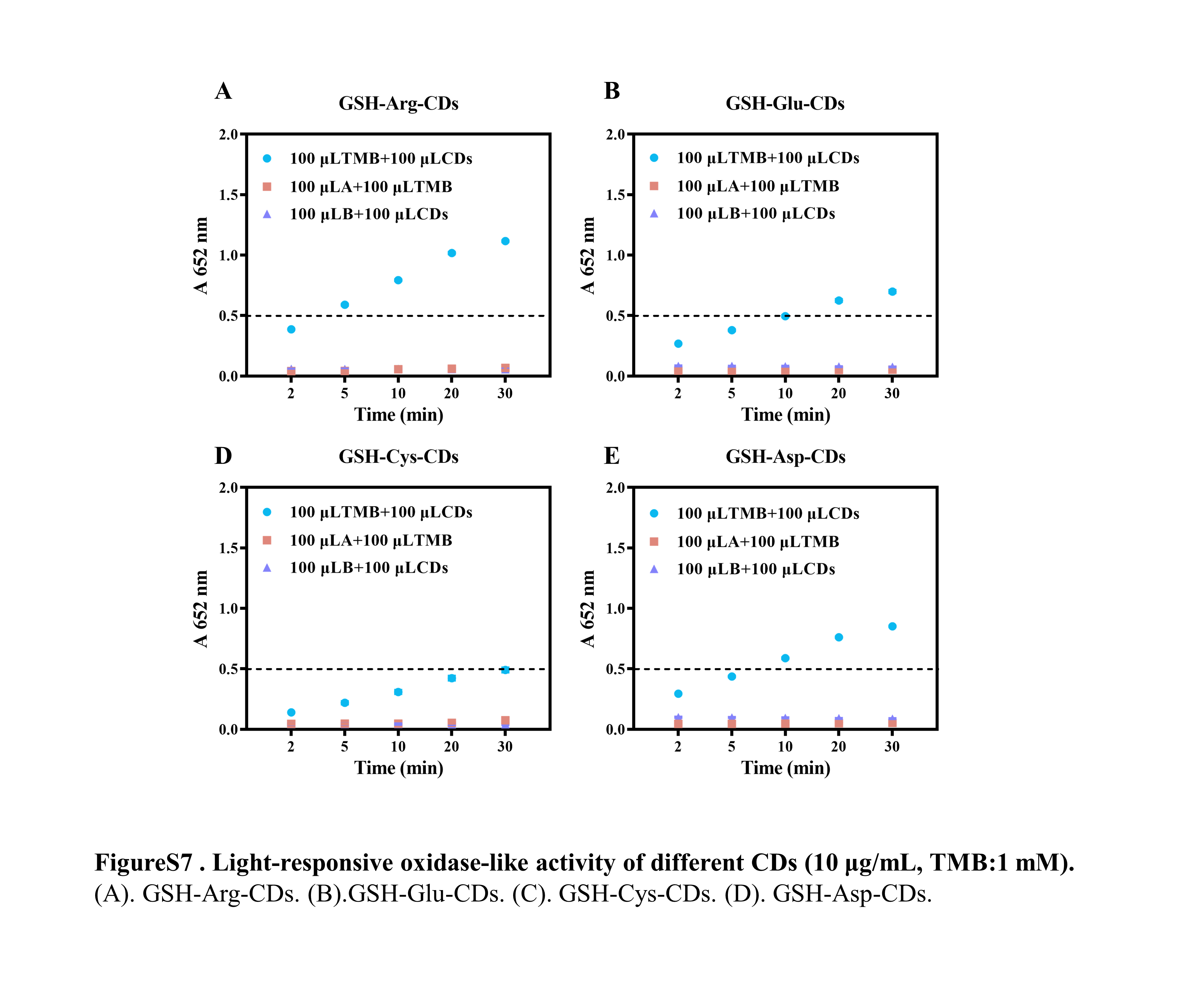


**Figure S7.** The light-responsive oxidase-like catalytic activity of different CDs (10 μg/mL, TMB:1 mM, AB buffer). **(A)** GSH-Arg-CDs **(B)** GSH-Glu-CDs **(C)** GSH-Cys-CDs **(D)** GSH-Asp-CDs.


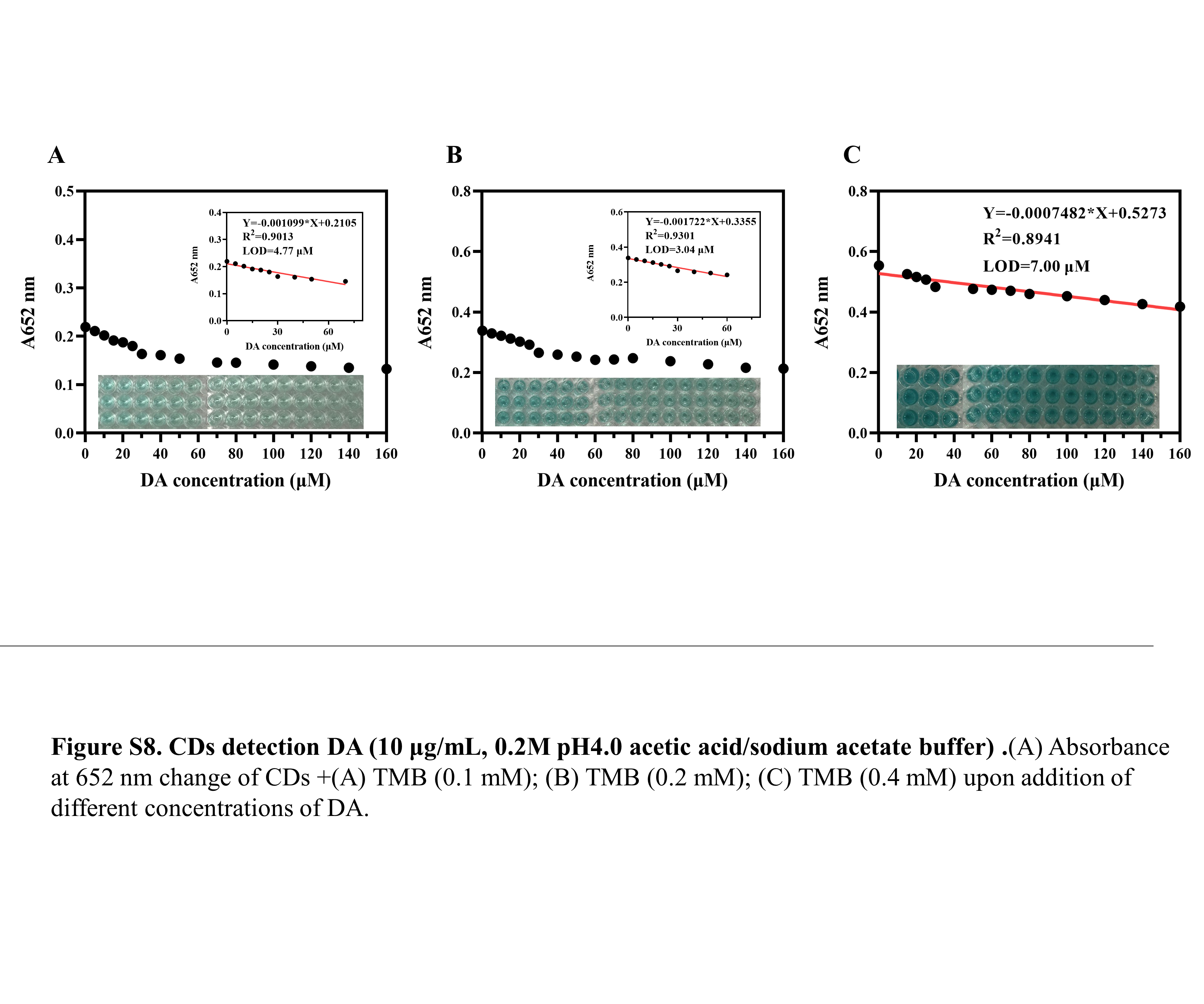


**Figure S8.** CDs detection DA (10 μg/mL, 0.2 M pH4.0 acetate buffer saline). The change in absorbance at 652 nm of CDs + **(A)** TMB (0.1 mM); **(B)** TMB (0.2 mM); **(C)** TMB (0.4 mM) upon addition of different concentrations of DA.

**Table S1**.

Comparison of the synthesized light-responsive CDs with other reported oxidase-like nanozymes.

|  | V_max_ | K_m_ | Reference |
| --- | --- | --- | --- |
| Fe-N-C | 0.601 nM S^-1^ | 1.81 mM | Gao et al., 2007 |
| Au QDs |  | 0.38 mM | Nagvenkar and Gedanken, 2016 |
| V_2_O_5_ | 1.85×10^-5^ M S^-1^ | 0.738 mM | Sun et al., 2016 |
| Cu_0.89_Zn_0.11_O | 2.877×10^-8^ M S^-1^ | 0.01 M | Singh et al., 2017 |
| NSC/Co1-xS |  | 2.98 mM | Wu et al., 2019 |
| Mn/Fe-MIL (53)-2 |  | 0.34 mM | Hu et al., 2021 |
| AuAgPd | 0.55×10^-8^ M s^-1^ | 1.22 mM | Kong et al., 2021 |
| Ps-Pt |  | 0.3742 mM | Ahmed et al., 2022 |
| Pd@AuNR | 29.75×10^-8^ M s^-1^ | 0.34 mM | Li et al., 2022 |
| HRP | 10.00×10^-8^ M s^-1^ | 0.434 mM | Luo et al., 2022 |
| CDs | 1.29 × 10^-8^ M s^-1^ | 0.35 mM | This work |

Ahmed, S.R., Sherazee, M., Srinivasan, S., and Rajabzadeh, A.R. (2022). Positively Charged Gold Quantum Dots: An Nanozymatic “Off-On” Sensor for Thiocyanate Detection. *Foods* 11, 1189. doi: 10.3390/foods11091189

Gao, L., Zhuang, J., Nie, L., Zhang, J., Zhang, Y., et al., (2007). Intrinsic peroxidase-like activity of ferromagnetic nanoparticles. *Nat. Nanotechnol.* 2**,** 577-583. doi: 10.1038/nnano.2007.260

Hu, J., Tang, F., Wang, L., Tang, M., Jiang, Y.-Z., et al., (2021). Nanozyme sensor based-on platinum-decorated polymer nanosphere for rapid and sensitive detection of Salmonella typhimurium with the naked eye. *Sens. Actuators, B* 346, 130560. doi: 10.1016/j.snb.2021.130560

Kong, J., Zheng, J., Li, Z., Huang, J., Cao, F., et al., (2021). One-pot synthesis of AuAgPd trimetallic nanoparticles with peroxidase-like activity for colorimetric assays. *Anal. Bioanal. Chem.* 413**,** 5383-5393. doi: 10.1007/s00216-021-03514-1

Li, J., Liu, T., Dahlgren, R.A., Ye, H., Wang, Q., et al., (2022). N, S-co-doped carbon/Co1-xS nanocomposite with dual-enzyme activities for a smartphone-based colorimetric assay of total cholesterol in human serum. *Anal. Chim. Acta* 1204, 339703. doi: 10.1016/j.aca.2022.339703

Luo, L., Ou, Y., Yang, Y., Liu, G., Liang, Q., et al., (2022). Rational construction of a robust metal-organic framework nanozyme with dual-metal active sites for colorimetric detection of organophosphorus pesticides. *J. Hazard. Mater.* 423, 127253. doi: 10.1016/j.jhazmat.2021.127253

Nagvenkar, A.P., and Gedanken, A. (2016). Cu0.89Zn0.11O, A New Peroxidase-Mimicking Nanozyme with High Sensitivity for Glucose and Antioxidant Detection. *ACS Appl. Mater. Interfaces* 8**,** 22301-22308. doi: 10.1021/acsami.6b05354

Singh, S., Tripathi, P., Kumar, N., and Nara, S. (2017). Colorimetric sensing of malathion using palladium-gold bimetallic nanozyme. *Biosens. Bioelectron.* 92**,** 280-286. doi: 10.1016/j.bios.2016.11.011

Sun, J., Li, C., Qi, Y., Guo, S., and Liang, X. (2016). Optimizing Colorimetric Assay Based on V2O5 Nanozymes for Sensitive Detection of H2O2 and Glucose. *Sensors* 16, 584. doi: 10.3390/s16040584

Wu, Y., Jiao, L., Luo, X., Xu, W.Q., Wei, X.Q., et al. (2019). Oxidase-Like Fe-N-C Single-Atom Nanozymes for the Detection of Acetylcholinesterase Activity. *Small* 15, 1903108. doi: 10.1002/smll.201903108

**Table S2**

Comparison of performances among different nanozyme-based colorimetric DA detection methods

| Materials | Linear range (μM) | LOD (μM) | Nanozymes | Ref. |
| --- | --- | --- | --- | --- |
| Pt600-GLP NCs | 1-100 | 0.66 | Peroxidase | Lai et al., 2021 |
| h-CuS NCs | 2-150 | 1.67 | Peroxidase | Zhu et al., 2019 |
| Pt/CoSn(OH)_6_ | 5-60 | 0.76 | Peroxidase | Liu et al., 2019 |
| CoFe_2_O_4_/CoS hybrid nanotubes | 0-50 | 0.58 | Peroxidase | Yang et al., 2018 |
| PNPG-PEG | 5.1-125 | 4.6 | Peroxidase | Razavi et al., 2022 |
| Pt/hBNNSs-5 | 2-55 | 0.76 | Peroxidase | Ivanova et al., 2019 |
| NiCo_2_S_4_-rGO | 0.5-100 | 0.42 | Oxidase | Wang et al., 2018 |
| nanoceria | 1-800 | 1.5 | Oxidase | Hayat et al., 2015 |
| CDs | 0.5-15 | 0.25 | Light-responsive Oxidase | This work |

Lai, X., Han, Y., Zhang, J., Zhang, J.-Y., Lin, W.-F., et al. (2021). Peroxidase-Like Platinum Clusters Synthesized by Ganoderma lucidum Polysaccharide for Sensitively Colorimetric Detection of Dopamine. *Molecules* 26 (9), 2738. doi: 10.3390/molecules26092738

Zhu, J.-L., Peng, X., Nie, W., Wang, Y.-J., Gao, J.-W., et al. (2019). Hollow copper sulfide nanocubes as multifunctional nanozymes for colorimetric detection of dopamine and electrochemical detection of glucose. *Biosens. Bioelectron.* 141, 111450. doi: 10.1016/j.bios.2019.111450

Liu, H., Ding, Y.-N., Bian, B., Li, L., Li, R., Zhang, X., et al. (2019). Rapid colorimetric determination of dopamine based on the inhibition of the peroxidase mimicking activity of platinum loaded CoSn(OH)_6_ nanocubes. *Microchim. Acta* 186 (12), 755. doi: 10.1007/s00604-019-3940-5

Yang, Z., Zhu, Y., Chi, M., Wang, C., Wei, Y., and Lu, X. (2018). Fabrication of cobalt ferrite/cobalt sulfide hybrid nanotubes with enhanced peroxidase-like activity for colorimetric detection of dopamine. *J. Colloid. Interface Sci.* 511, 383-391. doi: 10.1016/j.jcis.2017.09.097

Razavi, M., Barras, A., Szunerits, S., Khoshkam, M., Kompany-Zareh, M., and Boukherroub, R. (2022). A colorimetric assay and MCR-ALS analysis of the peroxidase-like activity of poly (N-phenylglycine) functionalized with polyethylene glycol (PNPG-PEG) nanozyme for the determination of dopamine. *Anal. Chim. Acta* 1235,340493. doi: 10.1016/j.aca.2022.340493

Ivanova, M.N., Grayfer, E.D., Plotnikova, E.E., Kibis, L.S., Darabdhara, G., Boruah, P.K., et al. (2019). Pt-Decorated Boron Nitride Nanosheets as Artificial Nanozyme for Detection of Dopamine. *Acs Appl. Mater.* *Interfaces* 11 (25), 22102-22112. doi: 10.1021/acsami.9b04144

Wang, Y., Yang, L., Liu, Y., Zhao, Q., Ding, F., Zou, P., et al. (2018). Colorimetric determination of dopamine by exploiting the enhanced oxidase mimicking activity of hierarchical NiCo_2_S_4_-rGO composites. *Microchim. Acta* 185 (10), 496. doi: 10.1007/s00604-018-3035-8

Hayat, A., Cunningham, J., Bulbul, G., and Andreescu, S. (2015). Evaluation of the oxidase like activity of nanoceria and its application in colorimetric assays. *Anal. Chim.* *Acta* 885, 140-147. doi: 10.1016/j.aca.2015.04.052
